# Monitoring of a Highly Flexible Aircraft Model Wing Using Time-Expanded Phase-Sensitive OTDR

**DOI:** 10.3390/s21113766

**Published:** 2021-05-28

**Authors:** Miguel Soriano-Amat, David Fragas-Sánchez, Hugo F. Martins, David Vallespín-Fontcuberta, Javier Preciado-Garbayo, Sonia Martin-Lopez, Miguel Gonzalez-Herraez, María R. Fernández-Ruiz

**Affiliations:** 1 Departamento de Electrónica, Universidad de Alcalá, Alcalá de Henares, 28805 Madrid, Spain; david.fragas@edu.uah.es (D.F.-S.); sonia.martinlo@uah.es (S.M.-L.); rosario.fernandezr@uah.es (M.R.F.-R.); 2 Aragon Photonics Labs (APL), C/Prado 5, 50009 Zaragoza, Spain; j.preciado@aragonphotonics.com; 3 Instituto de Óptica, CSIC, 28006 Madrid, Spain; hugo.martins@csic.es; 4 R&D Department, ASD Division, Capgemini Engineering, C/Campezo 1, 28022 Madrid, Spain; david.vallespinfontcuberta@altran.com

**Keywords:** structural health monitoring, aircraft, flexible wings, time-expanded-ΦOTDR, Rayleigh scattering, dual frequency combs

## Abstract

In recent years, the use of highly flexible wings in aerial vehicles (e.g., aircraft or drones) has been attracting increasing interest, as they are lightweight, which can improve fuel-efficiency and distinct flight performances. Continuous wing monitoring can provide valuable information to prevent fatal failures and optimize aircraft control. In this paper, we demonstrate the capabilities of a distributed optical fiber sensor based on time-expanded phase-sensitive optical time-domain reflectometry (TE-ΦOTDR) technology for structural health monitoring of highly flexible wings, including static (i.e., bend and torsion), and dynamic (e.g., vibration) structural deformation. This distributed sensing technology provides a remarkable spatial resolution of 2 cm, with detection and processing bandwidths well under the MHz, arising as a novel, highly efficient monitoring methodology for this kind of structure. Conventional optical fibers were embedded in two highly flexible specimens that represented an aircraft wing, and different bending and twisting movements were detected and quantified with high sensitivity and minimal intrusiveness.

## 1. Introduction

Structural health monitoring (SHM) [1] has become a topic of intense research, nowadays. The early detection of cracks and failures in critical structures like bridges, pipelines or aircrafts can prevent substantial material and personal damage. In particular, in the case of aircraft, there is currently a trend towards highly flexible wings, aimed at ensuring high-efficiency, long-endurance aircraft with improved fuel consumption. However, the different loads on the wings, mainly aerodynamic, inertial and propulsive, generate structural torsion and bending in flight, which are particularly acute in this type of wings [2]. Knowing the deformation limits as well as the evolution of the wing shape is of crucial importance for designers. Moreover, aerodynamically induced vibration in the wings, coupled with the structural modes of oscillation can result in fatal consequences for the integrity of the vehicle. This phenomenon is called flutter. The continuous monitoring of this kind of structure with sufficiently high resolution would require a high number of sensors, with cumbersome circuitry and bulkiness. Optical fiber sensors (OFS) represent a cost-effective solution with strong potential for this particular application. In particular, OFS stand out for their reduced weight, small size, immunity to electromagnetic interference and the possibility to perform multiplexed sensing in a single optical fiber [1,3]. A widespread OFS solution is that of punctual sensors based on fiber Bragg gratings (FBGs) [4,5]. FBGs can be understood as all-optical filters since their structure (a periodic variation of the fiber effective refractive index) reflects certain wavelengths within a bandwidth around the Bragg wavelength (related to the refractive index period), while the rest are transmitted. The Bragg wavelength is highly dependent on the environmental conditions around the fiber. Mechanical and thermal perturbations on the fiber produce a proportional shift on the grating reflection wavelengths, this being the principle of measurement. The interrogation of several FBGs written in a single optical fiber is made possible by time and/or wavelength multiplexing [4,6,7]. Despite theoretical studies that affirm the possibility of multiplexing up to one thousand FBGs [8], in practical terms, the multiplexing of hundreds of FBGs represents a challenge [6,9]. Besides, the FBGs have to be precisely written in the fiber, increasing the cost and complexity of such sensors. When a high number of sensing points (>100) are required, distributed optical fiber sensors (DOFS) represent a suitable solution for SHM, since all the segments of the fiber act as sensing units, only separated by the attainable spatial resolution [10,11]. Distributed sensors are being employed for monitoring dams [12], bridges [13], pipelines [14,15] or blades [16,17]. Optical frequency domain reflectometry (OFDR) based on Rayleigh scattering delivers high spatial resolution, reaching submillimetric resolution in tens of meters [18,19]. The optical source in OFDR is a tunable laser source that must be linearly frequency shifted without mode hops. Attaining a high linear frequency sweep is a challenging task, which typically implies a low laser sweep rate, thus hindering the possibility of performing dynamic measurements. Besides, the effect of the small residual nonlinearities in the sweep have to be minimized in order to achieve high-resolution [19]. Various solutions have been developed to address this constraint, including the incorporation of an external interferometer, as well as an interpolation algorithm to correct the frequency sampling. However, applied solutions typically lead to an increase in the requirements of the processing system, as well as a limitation in the ability to change the measurement range. On the other hand, phase-sensitive optical time-domain reflectometry (ΦOTDR) configurations usually offer ranges of tens up to more than 100 km (i.e., assisted by distributed amplification schemes, such as Raman amplification [20,21]) with a spatial resolution of several meters. The acoustic sampling in ΦOTDR schemes is only limited by the length of the fiber, being capable of measuring dynamic variations up to the MHz regime [22]. Spatial resolution of a few centimeters can be achieved by using coding techniques, although substantially increasing the detection and acquisition bandwidths is linked to higher cost and power dissipation [23]. Furthermore, the required decoding algorithms in this case, along with the high amount of data generated, implies a heavy computational load that also prevents real-time measurement in most cases.

Very recently, the authors have proposed a novel time-expansion (TE-)ΦOTDR scheme [24], capable of interrogating fibers with cm spatial resolution and low bandwidth electronics (well below the MHz). This system interrogates the fiber by using a dual optical frequency comb generated from a low phase noise laser and electro-optical modulators. While one comb is sent to the fiber, the other comb (almost identical to the first one but with slightly different line spacing) is employed as a reference for performing a multiheterodyne detection [24]. The beating of the two combs leads to an effective “time expansion” of the recovered time signal, corresponding to compression in the frequency domain. Typical time-expansion factors can range between 3 to 5 orders of magnitude. Hence, it is possible to acquire a signal with a few GHz optical bandwidth (corresponding to cm resolution) by employing low bandwidth electronics of hundreds or even tens of kHz (depending of the selected line spacing offset of the dual comb). In the proposed TE-ΦOTDR configuration, an identical phase spectral coding is introduced in the two combs. This entails a significant improvement of the signal to noise ratio (SNR) of the retrieved signal while avoiding high-peak-power pulses, with no need for any further decoding. The reason is that, upon detection, the phase coding imposed on the probe and reference combs are cancelled out. The novel TE-ΦOTDR scheme rises as a cost-effective DOFS, granting very high-resolution and real-time monitoring.

In this paper, we present, for the first time to our knowledge, the first proof of concept demonstration of SHM of a highly flexible aircraft wing using a high spatial resolution ΦOTDR system. The TE-ΦOTDR depicted previously is used to interrogate two extruded polystyrene (XPS) specimens with an optical fiber embedded into them. The specimens are designed to simulate the stresses to which a common flexible wing is subjected. The high resolution of the system allows us to monitor how the specimens are affected by the different mechanical perturbations applied.

## 2. Materials and Methods

### 2.1. Principle of Operation of the TE-ΦOTDR System

In this section, we briefly outline the operation principle of the TE-ΦOTDR technique. TE-ΦOTDR combines the traditional Φ-OTDR with concepts from dual frequency comb spectroscopy [25]. In the TE-ΦOTDR scheme, an optical frequency comb is sent to the fiber for interrogation. The electrical field of this probe comb can be mathematically expressed as:

(1) $$e_{p}\left(t\right)=\sum_{m=0}^{N}A_{m}\exp\left[j\left(2\pi \upsilon_{m}t+\phi_{t}\right)\right],$$

where $A_{m}$, $\upsilon_{m}$ and $\phi_{m}$ are the amplitude, frequency and phase of the *m*th comb line, and N is the number of lines. The amplitude of all the lines is identical, $A_{m}=A,\;\forall \;m$. The frequency of each line can be decomposed in terms of the repetition rate ∆f, i.e., $\upsilon_{m}=\upsilon_{0}+m\cdot \Delta f$, with $\upsilon_{0}$ the frequency of the lowest frequency line. The probe frequency comb has a random spectral phase modulation so that the optical power is distributed across the full interrogation time (significantly increasing the average power sent into the fiber). In the frequency domain, the frequency comb can be simply described by a series of Dirac delta functions,

(2) $$E_{p}\left(f\right)=F\left\{e_{p}\left(t\right)\right\}=\sum_{m=0}^{N}A\cdot e^{j\phi_{t}}\cdot \delta \left(f-\upsilon_{m}\right),$$

where F{·} represents Fourier transformation. This probe comb is launched to the fiber under test (FUT), which has an impulse response b(t). The backscatterd light is the result of the spectral sampling of b(t) by the comb:

(3) $$E_{bs}\left(f\right)=\sum_{m=0}^{N}A\cdot \left|B\left(\upsilon_{m}\right)\right|\cdot e^{j\left(\phi_{t}+∠B\left(\upsilon_{m}\right)\right)}\cdot \delta \left(f-\upsilon_{m}\right),$$

with B(f)=F{b(t)}. The backscattered light is beaten with a second comb (acting as local oscillator, LO) almost identical to the probe, but with slightly different line spacing,

(4) $$E_{LO}\left(f\right)=\sum_{m=0}^{N}A\prime \cdot e^{j\phi_{t}}\cdot \delta \left(f-\upsilon \prime_{m}\right),$$

where $\upsilon \prime_{m}=\upsilon \prime_{0}+m\cdot \Delta f\prime$, with $\Delta f^{\prime }=\Delta f+\delta f$ and the line spacing offset δf≪Δf. Upon detection of the beating between the backscattered light and the LO comb, we obtain a series of radio-frequency (RF) combs. By filtering in the lines arising from the neighboring pair of lines (i.e., the lowest frequency lines below Δf/2), we obtain a single RF comb whose voltage in frequency domain is

(5) $$V\left(f\right)=A\cdot A^{\prime }\cdot \sum_{m=0}^{N}\left|B\left(\upsilon_{m}\right)\right|\cdot e^{j∠B\left(\upsilon_{m}\right)}\cdot \delta \left(f-\upsilon_{m}^{RF}\right),$$

where $\upsilon_{m}^{RF}=\left(\upsilon_{0}-\upsilon \prime_{0}\right)+m\cdot \delta f$. Note that the use of the same spectral phase in the probe and LO combs ensures that the obtained optical signal is directly the impulse response of the fiber in reflection, with no need for further demodulation processing. Additionally, the detected comb has been efficiently downconverted onto the RF domain. In this way, a broad optical bandwidth ($B_{o}=N\cdot \Delta f$, e.g., of few GHz, providing cm range resolutions) can be measured from a very narrow RF comb ($B_{e}=N\cdot \delta f$, e.g., under the MHz), with measurement bandwidth compression factors CF=Δf/δf. To be able to isolate the first-order beating frequencies between pairs of lines of the dual comb without aliasing, the maximum separation between pairs of lines (i.e., N·δf) must be smaller than half the line spacing. To accomplish this condition, the offset between combs must satisfy the condition

(6) $$\delta f<\Delta f^{2}/\left(2B_{o}\right).$$

### 2.2. Experimental Setup

**Figure 1:**
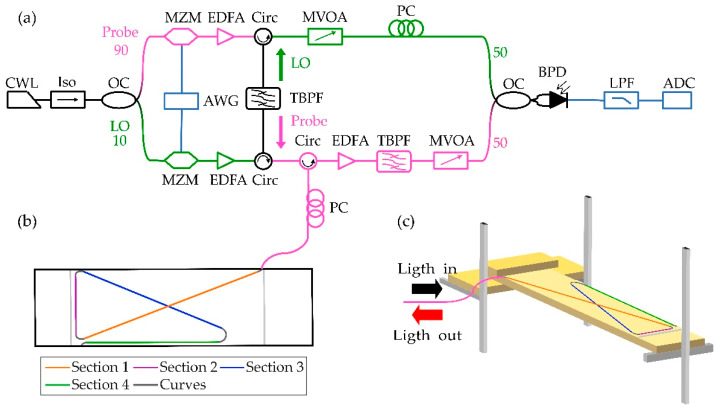
Experimental setup. (**a**) Diagram of the optical setup to carry out the measurements; (**b**) diagram of a representative specimen with a sensing optical fiber embedded following a cross topology complemented with a parallel bar; (**c**) diagram of a specimen placed on the test bench used in the test. OC: optical coupler; Iso.: isolator; Circ.: circulator; PC: polarization controller. MVOA: manual variable optical attenuator. The rest of the acronyms are described in the text.

The setup employed for performing the experiments is shown in Figure 1. A narrow linewidth continuous wave laser (CWL, NKT Koheras X15, centered at 1550.12 nm, with a linewidth < 100 Hz) was used to seed two Mach Zehnder modulators (MZM, Photline MX-LN-10, Oclaro SD-20). The modulators were driven by an arbitrary waveform generator (AWG, Keysight M8195A, 32 GSa/s and analog bandwidth of 12.8 GHz) that reproduced a modulation signal designed offline with the comb information [26]. It should be noted that these RF waveforms can be generated by other cheaper solutions, e.g., based on field-programable gate arrays (FPGAs) and digital-to-analog converters. Since the AWG only reproduced a real-valued signal, the spectrum of each modulated signal was composed of two sidebands around the laser frequency. As shown in Figure 1a, the upper branch generated the probe comb while the lower branch generated the LO comb. Both combs were boosted by two erbium-doped fiber amplifiers (EDFAs). To ensure an unambiguous down-conversion, one sideband of each modulated signal was suppressed by means of a tunable band-pass filter (TBPF, Yenista XTM-50). This filter also removed the amplified spontaneous emission (ASE) introduced by the EDFAs and the residual contributions of the carrier. The probe signal was then launched to the fiber under test embedded in the XPS specimen (see Section 2.3). The backscattered signal was amplified by another EDFA and properly filtered by another TBPF to reduce the ASE. The LO and probe signals were then beaten by means of balanced photodetector (BPD, Thorlabs BPD410C, 100 MHz) and digitized by an analog to digital converter card (ADC, NI PXIE 5122, 100 MSa/s). An electrical low-pass filter (LPF, 2 MHz) was employed to reduce the sampling requirements of the ADC.

### 2.3. Specimen Description

As previously mentioned, the development of highly flexible, lightweight wings is sought after in the aeronautic field. The use of less structurally rigid wings could be critical to future long-range, fuel-efficient airliners. To analyze the validity of the TE-ΦOTDR-based distributed optical fiber sensors for this application, the flexible wings were modelled with rectangular specimens of extruded polystyrene material (XPS). The specimens had an aspect ratio similar to that employed in an unmanned aerial vehicle (UAV). Two specimens with different thicknesses were used: specimen A, with dimensions 99.35 cm × 20 cm × 3 cm; and specimen B, with approximately the same area but three times thinner, i.e., with dimensions 100.8 cm × 20 cm × 1 cm.

To embed the fiber in the specimen, a small groove was made in each sample, following the shape depicted in Figure 1b. In this figure, four different linear sections were indicated. The fiber bends had a sufficiently high curvature radius (2.6 cm) to ensure minimal macrobending losses. This fiber was unjacketed but maintaining the acrylate coating. The fiber had been manually tensioned and coated with a layer of commercial epoxy glue (Loctite 3430). A safety margin was left at the longitudinal extremes of the specimens to allow the specimen installation in the test bench (Figure 1c). The total fiber length was about 8.5 m, from which 2 m were embedded in the specimen.

## 3. Results

In this paper, three different kinds of stress were applied to the specimens, namely bending, torsion, and vibration. These three phenomena were selected as representative examples of the uncoupled effects suffered by aircraft wings throughout the different stages of flight. For the experiments, the dual frequency combs consisted of 1000 lines with a line spacing of 5 MHz and an optical bandwidth of 5 GHz. In practical terms, this means a measurement range of 20 m and a spatial resolution of 2 cm. The line spacing difference between the two combs, which corresponded to the sensor’s acoustic sampling, was set at 80 Hz.

### 3.1. Bending

**Figure 2:**
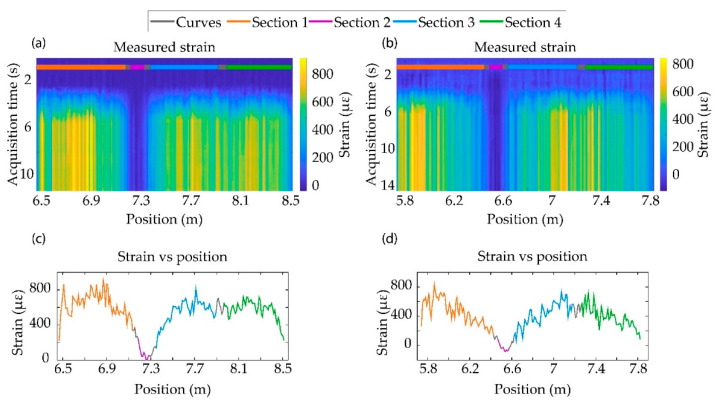
Plots of flexion test results: (**a**,**b**) represent the value of the strain measured at the different positions of the FUT at different acquisition instants on specimens A and B, respectively; (**c**,**d**) represent the mean value of the deformation between the acquisition instants 6–8 s at each position of the FUT in samples A and B, respectively.

To perform the bending test, one end of the specimen was fixed on the test bench (see Figure 1c). The other end was free, so that we could apply a perpendicular force to flex the wing. Since the glued fiber was joined with the movements of the specimens, this flexion process caused a stress on the optical fiber. The strain map evolution when perpendicular forces were applied to specimens A and B are shown in Figure 2a,b, respectively. The experiment started with the sample in the resting state, so the strain remained constant during the first moments of the test. However, when the specimens were flexed, a positive strain was detected in sections 1, 3 and 4. The reason was that the bending of the specimens caused an elongation of the fiber that was glued to the top surface of the material. The flexion was maintained during a few seconds, in order to see the temporal stability of our measurements.

The curvature suffered in specimen A (the thicker one, in Figure 2c) was more pronounced closer to the test bench, while in further positions where the fiber was deployed, the radius of curvature was lower. This result was reflected in the measured strain on the fiber, since the strain value recorded in sections 1, 3 and 4 decreased in the fiber sections furthest away from the test bench. In specimen B (Figure 2d), however, the whole specimen suffered a curvature whose radius linearly varied along its length, being again more pronounced closer to the test bench. The recorded values of strain showed a higher fiber elongation closer to the test bench, while it lessened linearly up to reaching the farthest position. The recorded strain was symmetrical in the cross topology (sections 1 and 3), as expected. In the longitudinal fiber section (section 4), we also observed a linear variation of strain with a practically identical slope, in good agreement with what was expected, due to the fiber topology. In all cases, section 2 did not suffer from any strain, as it was perpendicular to the applied force.

An acute ripple was observed in the measurements. This could be attributed to the manual glue of the fiber on the specimen, and the different density of glue substance in the groove along the fiber length, readily causing dissimilar strain transfer to the fiber. 

### 3.2. Torsion

In the torsion test, one corner of the free end of the specimen was fixed to the test bench, leaving the other one released to exert the torsional stress. In specimen A, the left corner was fixed, while a downward force was applied in the other corner to torsion the specimen. However, in specimen B, the right corner was blocked, and the left one was pushed upwards to deform the specimen.

**Figure 3:**
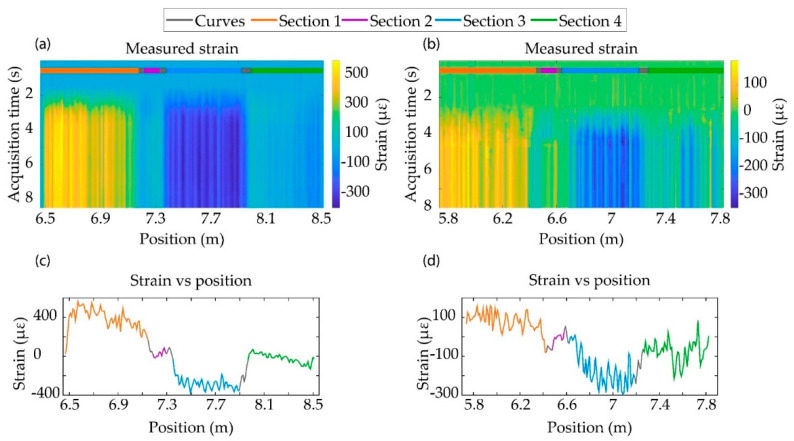
Plots of torsion test results; (**a**,**b**) represent the value of the strain measured at the different positions of the FUT at each acquisition instant on specimens A and B, respectively; (**c**,**d**) represent the mean values of the deformation between the acquisition instants 6–8 s at each position of the FUT in samples A and B, respectively.

As before, the sample started from the resting state. At a certain instant of time, torsion was performed and maintained for a few seconds. Due to the fiber distribution in the specimen, the experiment performed over the sample A caused a stretch in Section 1, producing a positive strain, as can be seen in Figure 3a in yellow (or orange tonalities in Figure 3c). However, the torsional movement produced a compression over Section 3, corresponding with blue tonalities in Figure 3a (or light blue section in Figure 3c). Despite the different processes to achieve torsion in the specimens, the movement to twist specimen B produced a compression and stretch process in the same way as in Specimen A. For this reason, the patterns of deformation shown in Figure 3b,d are the same as in Figure 3a,c, but with different absolute values of measured strain.

As before, the different curvatures of the specimen generated a non-homogeneous strain profile. Since specimen B was thinner, the recovered stress was lower, as can be seen in Figure 3d. The variability of the stress recovered could be caused, again, by the manual tension of the fiber and non-homogeneous distribution of the glue.

### 3.3. Vibration

**Figure 4:**
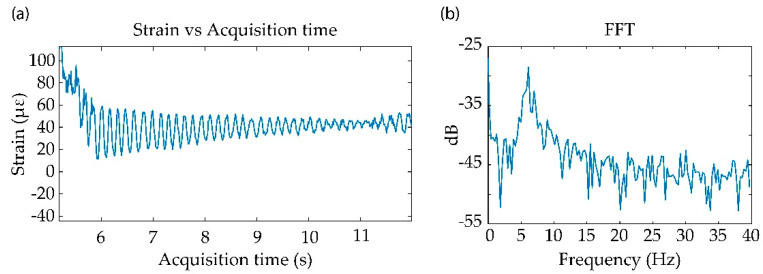
Vibration tests results. (**a**) Representation of the strain value measured at the different acquisition instants for the fiber position 6.98 m; (**b**) Fourier transform representation of the strain values of the vibration signal.

In this work, we have tested the validity of the sensing system to detect vibrations on the structure. To produce the vibration, the free end of the specimen was bent upwards and released, producing an oscillating movement up to return to the resting state. By tracking the temporal evolution of one stressed point (located at 6.98 m), we recovered a damped sinusoidal vibration as can be seen in Figure 4a. The spectral content of the perturbation is represented in Figure 4b, where it is estimated that the natural frequency of the vibration was approximately 6 Hz.

## 4. Discussion

In this proof of concept test, we have demonstrated the capability of the TE-ΦOTDR system of monitoring different mechanical processes in specimens replicating highly flexible aircraft wings. The high spatial resolution exhibited by the system allowed us to recover information from ~100 individual points distributed through the specimen’s surface. Besides, the use of low bandwidth electronics enabled the possibility of performing acoustic sensing in real-time in a highly spectral and cost efficient manner. Since the optical fiber was only sensitive to elongations, a smart topology of the embedded fiber has to be engineered to ensure that any kind of strain perturbation can be detected by at least one sensing fiber section. The selected topology in this case was a cross, as depicted in Figure 2c, which was further complemented with an additional section parallel to the specimen’s longitudinal axis. From a mathematical point of view, the strain measured by the fiber at each point was a consequence of projecting the specimen strain vector onto each fiber section. This can be seen in the recovered strain profile from the flexion and torsion experiments. For instance, in the flexion test, when the specimens were bent, sections 1, 3 and 4 are longitudinally stressed. Due to the angle formed between section 4 with respect to the sections 1 and 3, the strain measured by sections 1 and 3 decreases by a factor |cos(α)| with respect to Section 4, α being the angle between section 1 and section 4. For both specimens, cos(α) = 0.98, showing little difference in a practical viewpoint, in line with the values shown in Figure 2 and Figure 3. As expected, section 2 was insensitive to bending, as the flexion movement did not stress this fiber section. The strain profile of the twisted specimens followed a different pattern. In this case, sections 1 and 3 detected an elevated expansion and compression of the surfaces in which the fiber was embedded (with respect to the strain in the resting position), contrary to sections 2 and 4, which recovered small strain values. Hence, the torsion experiment shows the importance of the embedded fiber topology for recovering the information of the specimen deformation. 

In all the experiments, we can highlight how the variability of the strain value along the stressed sections allowed us to detect the nonuniform deformation of the specimen. In particular, it was apparent that the recorded strain values were dependent on the different radius of curvature of the specimen along its length. Hence, the distributed nature of the TE-ΦOTDR monitoring technique offers a useful tool to perform shape sensing of the flexible wing. A further study of this shape sensing capability remains as future work. On the other hand, we can observe high ripples in the detected strain curves. As previously commented, this can be attributed to the fact that the fiber was embedded manually, and hence, it had not been glued and tensioned uniformly. Therefore, the strain transfer was not homogeneous throughout the sensing fiber length. Other parameters such as groove depth and the specimen material can affect the strain information transference from the specimen surface to the fiber. For the first proof of concept, we chose XPS and commercial epoxy glue because of their availability and low cost. However, for future studies with real aeronautical materials, a strain transfer calibration should be performed to compensate the inhomogeneities mentioned above. Besides, in these measurements, we have experimentally validated the epoxy glue and the fact that the fiber was embedded in the XPS material act as thermal isolator, significantly reducing the effect of the temperature cross-sensitivity with respect to the released fiber.

Despite showing only the two meters embedded in the specimen, the experimental conditions would permit the attainment of a range of 20 m. That is, if we were to embed the entire fiber in the structure, we would have obtained information from 1000 independent points, maintaining the same spatial resolution, acoustic sampling and real-time measurement parameters shown above. Besides, according to Equation (6), it is possible to increase the acoustic sampling of the sensor up to 2.5 kHz, while maintaining the other parameters. For example, in a real system with the considered dimensions, the attained sensing performance would have permitted a single fiber to monitor the two wings plus the UAV fuselage with an acoustic sampling. This is a significant improvement over systems based on multiplexed FBGs, in which multiplexing over 100 gratings is a challenging task. FBGs, however, have been proven to deliver higher acoustic sampling, reaching in some cases 200 kHz, at the cost of reducing the sensitivity (see Table 1) [27]. This speed decreases notably with an increase of the number of FBG when performing quasi distributed sensing. As shown in Table 1, it is possible to measure 2000 individual points along 24 m with an acoustic sampling and sensitivity comparable to our measurements [28]. However, in TE-ΦOTDR the employed fiber is an off-the-shelf, untreated fiber, which provides a much simpler and cost-effective monitoring means with straightforward deployment. Besides, as previously mentioned, it should be noted that the sampling limitation of the here employed TE-OTDR can reach acoustic samplings in the kHz range. Regarding the distributed sensing techniques; OFDR schemes are capable of reaching high resolution, in the mm scale [29]. In general, these systems employ a tunable laser for interrogating the fiber. However, the nonlinearities of the laser sweep have to be compensated for by an external interferometer limiting the acoustic sampling. In the high-resolution mode (mm), the attainable sensitivity is reduced (see Table 1). Other solutions with cm spatial resolution offer better sensitivity at the expense of reducing the number of sensing points and acoustic sampling [30] or increasing notably the photodetector bandwidth [31].

## 5. Conclusions

Highly flexible wings in aerial vehicles are increasingly attracting the attention of researchers and engineers for their important advantages over traditional rigid structures. In this paper, we present a new methodology for the continuous monitoring of such wing structures, based on a novel distributed optical fiber sensor, namely, TE-ΦOTDR. TE-ΦOTDR stands out for its extraordinary high resolution (in the centimeter scale) and low detection and acquisition bandwidth needs, in contrast to traditional distributed sensing techniques. This technology has been tested in XPS structures (which model the structure of the wings of a UAV) of different thicknesses, in which an optical fiber has been bonded following a specifically engineered topology. Our experiments show that the technology is capable of performing a distributed detection of static (i.e., bending and torsion) and dynamic (i.e., vibration) mechanical deformations of the specimen. This is attained by obtaining the strain value suffered by the embedded optical fiber. In future studies, we will investigate the possibilities of performing shape sensing on the structure based on the recorded strain on the fiber topology, and we will move closer to a real situation, analysing different strategies for embedding the fiber in the real material of the UAV wings. Additionally, the strain coupling to the sample must be calibrated, allowing the measured strain values to be adjusted to improve the reliability of the measurements.

## Figures and Tables

**Table 1:** Comparison of different dynamic optical fiber sensor techniques with spatial resolution on the cm range.

Technology	Range	Sensitivity	Number of Sensing Points (Spat. Resol.)	Acoustic Samp	BW Photodtector
FBG (quasi distributed) [27]	24 m	1 με	2000	100 Hz	-
FBG [28]	-	75 με	214	200 kHz	-
OFDR [30]	30 m	200 nε	150 (20 cm)	50 Hz	≈MHz
OFDR [31]	950 m	100 nε	9500 (10 cm)	6.25 kHz	≈1 GHz
OFDR [29]	20 m	2 με	≈7700 (2.6 mm)	50 Hz	-
TE-ΦOTDR [24]	200 m	490 nε	10,000 (2 cm)	20 Hz	<1 MHz
TE-ΦOTDR (in this work)	20 m	1 με	1000 (2 cm)	80 Hz (max. 2.5 kHz)	<1 MHz

## Data Availability

The data presented in this study are available on request from the corresponding author.
